# Effectiveness of a Digital Falls Prevention (KOKU) to Improve Function and Reduce Falls Risk in Older Adults.

**DOI:** 10.1093/geroni/igaf122.4332

**Published:** 2025-12-31

**Authors:** Emma Stanmore, Amelia Parchment, Bolanle Odebiyi, Peter Bower, Chloe French, Chunhu Shi, Dawn Skelton, Chris Todd

**Affiliations:** University of Manchester, Stoke-on-Trent, England, United Kingdom; University of Manchester, Manchester, England, United Kingdom; University of Manchester, Manchester, England, United Kingdom; University of Manchester, Manchester, England, United Kingdom; University of Manchester, Manchester, England, United Kingdom; University of Manchester, Manchester, England, United Kingdom; Glasgow Caledonian University, Glasgow, Scotland, United Kingdom; University of Manchester, Manchester, England, United Kingdom

## Abstract

Functional decline and falls are common, challenging problems for older adults. Access and uptake to evidence-based exercise programmes are often sub-optimal. This study investigated the effectiveness of the KOKU digital strength and balance program for improving physical function and reducing falls risk among community-dwelling older adults. This two-arm, parallel group randomized controlled trial with an intention-to-treat data analysis enrolled eligible community-dwelling older individuals adults (60 years and older). Participants were randomized to either the intervention group comprising the digital strength and balance program (KOKU) alongside standard care (strength and balance exercise advice and falls prevention leaflet) or to the control group, receiving standard care only. The primary outcome measure was balance function (Berg Balance Score). Secondary trial outcomes included: lower limb strength; healthcare utilisation and health-related quality of life; concerns about falling; physical activity; falls risk, pain, mood, fatigue, self-reported falls, acceptability and usability of the KOKU program. 202 participants were recruited with a mean (SD) age of 76 (8.5) years, majority women (73%). At 3-month follow-up,181 participants (90%) had completed the trial. There was a statistically significant Berg Balance Score (mean difference 6.35 at 12-weeks, 95% CI, 4.48, 8.22), in favour of KOKU. Participants receiving the KOKU intervention had improved lower-limb strength as measured by the 5X Sit to Stand Chair test at 12-weeks compared to participants in the control group (mean difference: -6.85; 95% CI: -10.47, -3.24). The KOKU digital program could be considered as an effective self-management option to improve functional outcomes for older adults.

